# A Volumetric Waveguide-Type Rotman Lens Antenna for Three-Dimensional Millimeter-Wave Beamforming

**DOI:** 10.3390/s24092884

**Published:** 2024-04-30

**Authors:** Dong-Woo Kim, Soon-Soo Oh

**Affiliations:** 1 Radio Environment Safety Division, Radio Research Agency, 767, Bitgaram-ro, Naju-si 58323, Republic of Korea; dwkim91@korea.kr; 2 Department of Electronic Engineering, Chosun University, Gwangju 61452, Republic of Korea

**Keywords:** beamforming antenna, waveguide Rotman lens, three-dimensional beamforming

## Abstract

In this paper, a volumetric Rotman lens antenna operating at 28 GHz is proposed. The design formula and procedure were derived for the 3-D Rotman lens antenna. The number of tilted beams is 3 × 3. The six rectangular blocks are assembled using a metallic bolt. The input port consists of a waveguide, and the output port is made of an open-ended waveguide. The input and output waveguides are drilled in a flat conducting plate. The input and output port positions are optimized. Simulated and measured results show that the radiating beam is controlled almost exactly as calculated. Compared with the previous two-stage stacked Rotman lens antenna, the proposed Rotman lens antenna can dramatically decrease the antenna volume by approximately 75%.

## 1. Introduction

Fifth-generation (5G) new radio (NR) services have been commercialized, with an increasing need for high-rate and large-volume data links. Beamforming technology is essential for the successful service of 5G NR [1]. The analog beamforming technique is a good solution owing to its simple configuration and low cost compared with digital beamforming [2]. In particular, the Rotman lens was developed in 1963 [3], in which the beam direction is determined by choosing one of several input ports [3,4,5]. The Rotman lens is also used for generating multi-beam radiation [6,7,8,9]. Basically, a Rotman lens works as an array of the phase shifter.

In the early stages, the Rotman lens was implemented in the microstrip structure [10] due to its low cost and easy fabrication. However, in the high-frequency band, the waveguide type is more optimal because of the low loss [11,12]. The hybrid beamforming mixed with the analogue and digital beamforming also adopts the Rotman lens as the analog element [13].

However, the conventional Rotman lens is optimal for linear beam-tilting or beamforming. If the beam-tilting occurs in a two-dimensional direction, the structure of the Rotman lens should be a two-dimensional bulky structure. The Rotman lenses are connected in an interlaced form for dual-plane scanning: horizontal scan, vertical scan, or any combination of them [14,15]. A Rotman lens for a conformal plane was proposed, but the beam-tilting direction is along the cylindrical surface [16,17,18].

In this paper, a compact lens for three-dimensional beamforming is proposed. It is a volumetric waveguide type composed of six flat metallic blocks. The positions of the feeding waveguide and radiating elements were optimized. The frequency was set to 28 GHz for 5G applications [19].

## 2. Design Formula and Procedure of Three-Dimensional Rotman Lens

A general Rotman lens was fabricated after determining the focal arc F, the ratio of F to G, and the beam tilt angle α [3,4,5,6]. The previous design formula was two-dimensional, because the wavelength vector was in x,y coordinates [7,8,9,10]. Thus, a phase array antenna with a single Rotman lens can tilt the beam only through an angle of azimuth or elevation [3,4,5,6,7,8,9,10,11,12,13,14]. This paper presents the design of a three-dimensional Rotman lens. This allows for three-dimensional beamforming by varying the angles of both the azimuth and elevation. Moreover, the lens is compact.

The three-dimensional Rotman lens has three focal arcs, i.e., longitudinal, transverse, and diagonal arcs. A longitudinal or transverse Rotman lens can be designed using the general method. Longitudinal and transverse Rotman lenses designed using the previous formula are combined at the on-axis focal point G (−G,0,0) on the x-axis. The z-axis and the angle of the diagonal beam tilt must be considered when designing a diagonal Rotman lens.

**Figure 1:**
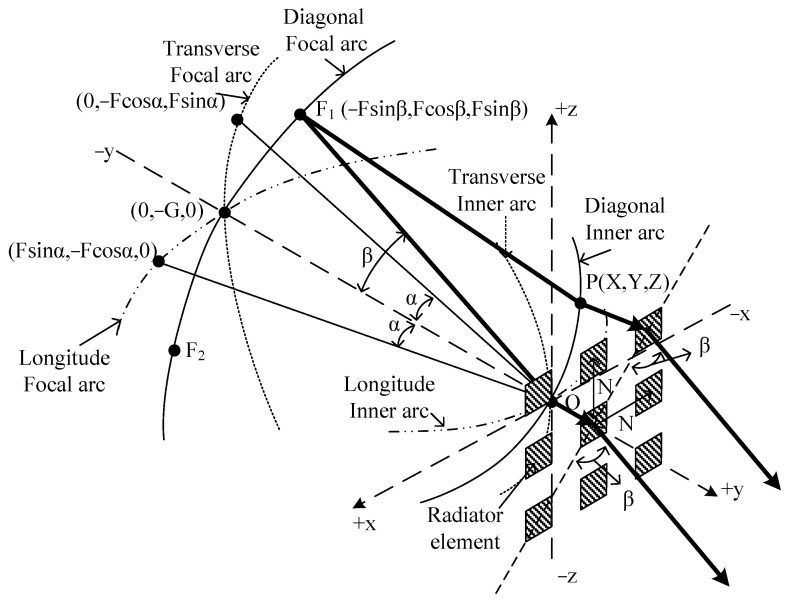
Schematic of three-dimensional waveguide Rotman lens.

A three-dimensional Rotman lens is shown in Figure 1. All wavelengths passing through the inner arc from any point on the focal arc must be the same. Vector formulae can be derived for the points F_1_ and F_2_ on the diagonal focal arc, as shown below. The vector formula assumes that the radiating elements are arranged in a square:

(1) $$\vec{F_{1}P}+W+\sqrt{2}N\sin\beta =F+W_{0\;\;}$$

(2) $$\vec{F_{2}P}+W-\sqrt{2}N\sin\beta =F+W_{0\;\;}$$

(3) $$\vec{GP}+W=G+W_{0}$$

Here, P is the point on the diagonal inner arc. The variables of W and W_0_ are the electrical length of the transmission between the P and the radiator element, where the subscript of 0 of W_0_ means the central port. The parameter F is the length between the central point and the longitude focal arc, respectively. The value of β is the angle between the central point and the diagonal focal arc. The distance N is the spacing between the radiating elements.

The squares of the vectors from F_1_, F_2_, and G on the diagonal focal arc to an arbitrary point, P, on the inner arc are given by (4), (5), and (6):

(4) $${\left(\vec{F_{1}P}\right)}^{2}=F^{2}+X^{2}+Y^{2}+Z^{2}+F^{2}{sin}^{2}\beta +2FX\cos\beta -2FY\sin\beta -2FZ\sin\beta$$

(5) $${\left(\vec{F_{2}P}\right)}^{2}=F^{2}+X^{2}+Y^{2}+Z^{2}+F^{2}{sin}^{2}\beta +2FX\cos\beta +2FY\sin\beta +2FZ\sin\beta$$

(6) $${\left(\vec{GP}\right)}^{2}={\left(G+X\right)}^{2}+Y^{2}+Z^{2}$$

Here, X, Y, and Z are the positions in the Cartesian coordinate on the diagonal inner-arc.

All parameters are normalized to F to develop an equation that defines the variables in terms of F, as follows:

$$\eta =\frac{N}{F},\;\;x=\frac{X}{F},y=\frac{Y}{F},\;\;z=\frac{Z}{F},\;\;g=\frac{G}{F},\;\;w=\frac{W-W_{0}}{F}$$

$$\alpha_{0}=\cos\alpha ,\alpha_{1}=\sin\alpha ,\beta_{0}=\cos\beta ,\beta_{1}=\sin\beta$$

The normalized variables are substituted into Equations (4)–(6) to yield (7)–(9):

(7) $${\left(\frac{\vec{F_{1}P}}{F}\right)}^{2}={\left(1-w-\sqrt{2}\eta \beta_{1}\right)}^{2}=1+w^{2}+2\eta^{2}{\beta_{1}}^{2}-2w-2\sqrt{2}\eta \beta_{1}+2\sqrt{2}w\eta \beta_{1}$$

(8) $${\left(\frac{\vec{F_{2}P}}{F}\right)}^{2}={\left(1-w+\sqrt{2}\eta \beta_{1}\right)}^{2}=1+w^{2}+2\eta^{2}{\beta_{1}}^{2}-2w+2\sqrt{2}\eta \beta_{1}-2\sqrt{2}w\eta \beta_{1}$$

(9) $$\frac{{\left(\vec{GP}\right)}^{2}}{F^{2}}={\left(g+x\right)}^{2}+y^{2}+z^{2}$$

When these normalized variables are substituted into (1)–(3), we obtain (10)–(12):

(10) $${\left(\frac{\vec{F_{1}P}}{F}\right)}^{2}={\left(1-w-\sqrt{2}\eta \beta_{1}\right)}^{2}=1+w^{2}+2\eta^{2}{\beta_{1}}^{2}-2w-2\sqrt{2}\eta \beta_{1}+2\sqrt{2}w\eta \beta_{1},$$

(11) $${\left(\frac{\vec{F_{2}P}}{F}\right)}^{2}={\left(1-w+\sqrt{2}\eta \beta_{1}\right)}^{2}=1+w^{2}+2\eta^{2}{\beta_{1}}^{2}-2w+2\sqrt{2}\eta \beta_{1}-2\sqrt{2}w\eta \beta_{1}$$

$$\vec{GP}=-W+W_{0}+G=-w+g$$

(12) $$\frac{{\left(\vec{GP}\right)}^{2}}{F^{2}}={\left(g-w\right)}^{2}=w^{2}-2gw+g^{2}=x^{2}+y^{2}+z^{2}+g^{2}+2gx$$

This is equivalent to subtracting (8) from (7) and (11) from (10); this removes x, as shown in (13), and we obtain a concise expression using only y and z:

(13) $$y+z=\sqrt{2}\eta \left(1-w\right)$$

If the radiating elements are arranged in a square, y is equal to z. With Equation (13), it is possible to define the values of y and z for an arbitrary point P as shown in (14):

(14) $$y=z=\frac{\sqrt{2}}{2}\eta \left(1-w\right)$$

This is the same as adding (8) to (7) and (11) to (10), which yields the simultaneous Equation (15). Moreover, Equations (9) and (12) yield (16), which can be solved using (15):

(15) $$x^{2}+y^{2}+z^{2}+{\beta_{1}}^{2}+2x\beta_{0}=w^{2}+2\eta^{2}{\beta_{1}}^{2}-2w$$

(16) $$\begin{array}{cc} \frac{{\left(\vec{GP}\right)}^{2}}{F^{2}}={\left(g-w\right)}^{2} & =w^{2}-2gw+g^{2}=x^{2}+y^{2}+z^{2}+g^{2}+2gx=w^{2}-2gw\\ & =x^{2}+y^{2}+z^{2}+2gx \end{array}$$

By subtracting (15) from (16), x is obtained as (17):

(17) $$x=\frac{2w-2gw-2\eta^{2}{\beta_{1}}^{2}+{\beta_{1}}^{2}}{2\left(g-\beta_{0}\right)}$$

Coefficients a–c can be obtained by substituting the determined x, y, and z into Equation (16), and then creating a quadratic equation of the formula $aw^{2}+bw+c=0$:

(18) $$a=\left(1-\eta^{2}\right)-{\left(\frac{1-g}{g-\beta_{0}}\right)}^{2}$$

(19) $$b=-2g-2g\left(\frac{1-g}{g-\beta_{0}}\right)-2\eta^{2}-{\beta_{1}}^{2}\frac{2\eta^{2}g-2\eta^{2}-g+1}{{\left(g-\beta_{0}\right)}^{2}}$$

(20) $$c=-g{\beta_{1}}^{2}\frac{1-2\eta^{2}}{g-\beta_{0}}-\eta^{2}-{\beta_{1}}^{4}{\left(\frac{\eta^{2}-\frac{1}{2}}{g-\beta_{0}}\right)}^{2}$$

These formulae aid the design of a three-dimensional, diagonal Rotman lens. The general formula consists of only x and y and the tilt angle α of the azimuth or elevation. When designing a three-dimensional Rotman lens, not only x and y, but also z, must be considered, as must the tilt angle β of the diagonal Rotman lens and the tilt angle α. We must obtain a quadratic equation for w of the diagonal Rotman lens using the parameters x, y, z, α, and β; parameters a-c must also be determined.

The three-dimensional Rotman lens is designed as follows:

First, define F, G, R, α, and β. Second, obtain coefficients a, b, and c of the quadratic equation w, which will aid in the design of a longitudinal or transverse Rotman lens in terms of point G on the x-axis. Third, design the focal and inner arcs for longitudinal and transverse Rotman lenses using the obtained x, y, and α. Fourth, obtain coefficients a–c of the quadratic equation w that is used to design a diagonal Rotman lens at point G on the x-axis. Fifth, design the focal and inner arcs of the diagonal Rotman lens using the obtained x, y, z, and β. Finally, combine all Rotman lens bases at point G on the x-axis.

## 3. Design and Simulation of Three-Dimensional Waveguide Rotman Lens

The longitudinal and transverse waveguides are positioned in relation to the designed focal arc, and the diagonal waveguides are positioned using the formula extracted in the previous section for that arc. The output phase of the waveguide at the inner lens is optimized, as is the input to the central waveguide. The waveguides are not the same length, because the outer surface of the Rotman lens is flat, and the lens is thus compact. All output waveguides of the inner lens must be of the same length to ensure constant phase differences between the waveguides. The length of a waveguide influences the phase, so the widths and heights of the waveguides are adjusted to match the guided wavelength of the output waveguide; the lengths are not adjusted.

**Figure 2:**
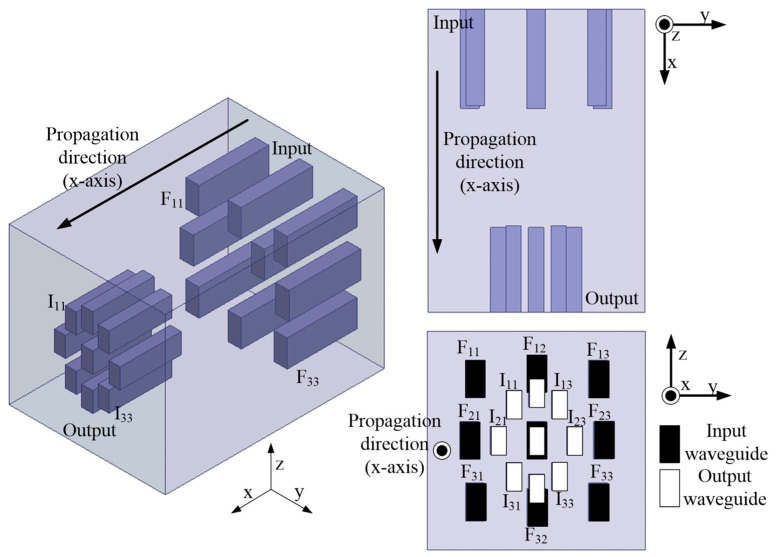
The simulation model for the three-dimensional waveguide Rotman lens.

**Figure 3:**
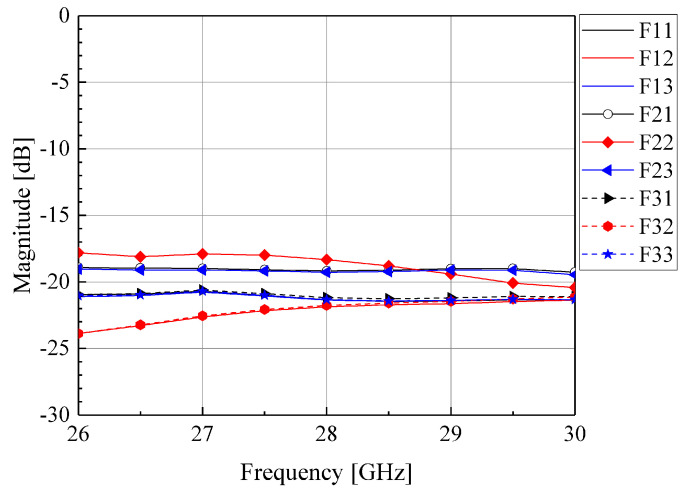
Simulation results of the transmission coefficients for a three-dimensional waveguide Rotman lens.

Figure 2 is the simulation model for the proposed three-dimensional waveguide Rotman Lens. Figure 3 shows the simulation results for the transmission coefficient from F_22_ of the central input waveguide to all output waveguides. Here, F_22_ means the index of the input port that is located in the center, as shown in Figure 2, and is different from reflection coefficient notions, such as S_22_. The simulation was performed using Ansys HFSS, i.e., a three-dimensional simulator [20]. The simulation featured nine input waveguides, nine output waveguides, and an open free space. The waves from one input waveguide that are incident on the focal arc are uniformly output at –18 to –23 dB to all ports at 28 GHz, although the transmission coefficient is not accurate given the loss to leakage outside the free space.

**Figure 4:**
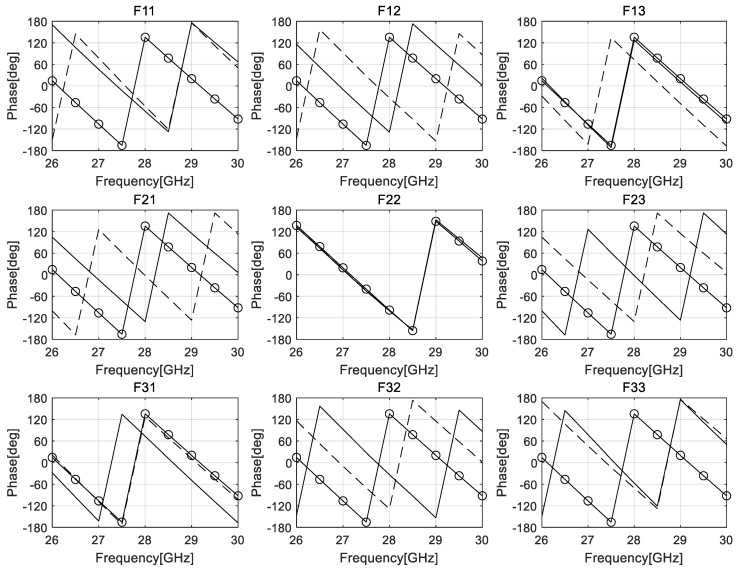
Simulation results for the phase of a three-dimensional waveguide Rotman lens. The subtitle denotes the input port. The dotted line, solid line, and symbol line are for output ports in the longitudinal, transverse, and diagonal direction, respectively.

The curves in Figure 4 show the phase differences between the focal arc input and the inner arc output. The three-dimensional Rotman lens consists of four two-dimensional Rotman lenses placed in the longitudinal and transverse directions, and at two diagonal positions, by reference to F_22_ of the central input port. The output phase differences between the focal and inner arcs of all two-dimensional Rotman lenses were analyzed.

Table 1 shows the simulation results at 28 GHz. The absolute values of the output phases from input ports F_21_ and F_23_, as shown in Figure 2 for the longitudinal Rotman lens, are 133°, as shown in Table 1. The output phases from inputs F_12_ and F_32_ of the transverse Rotman lens are 155°, i.e., 22° greater than that of the longitudinal lens. This yields a beam tilt angle of 2° to 3°. The absolute values of the output phases from inputs F_11_, F_13_, F_31_, and F_33_ of the diagonal Rotman lens are about 173°.

**Figure 5:**
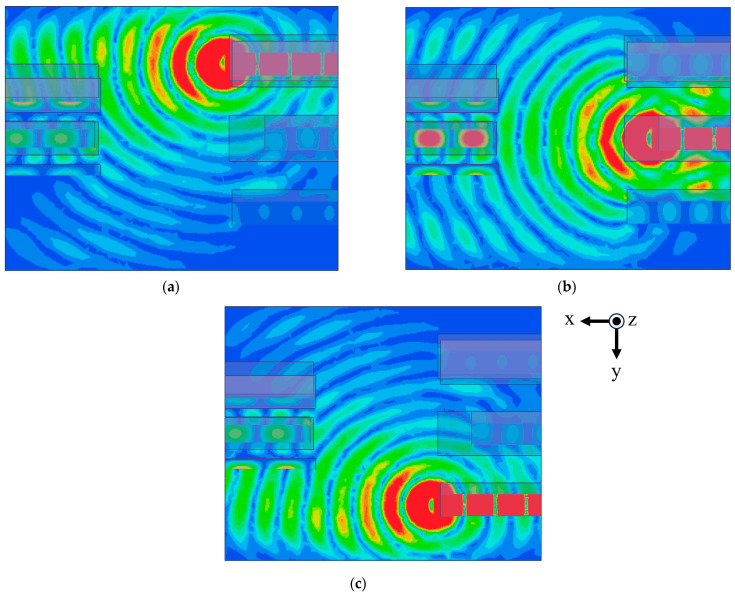
Simulation results for the electric field of a three-dimensional waveguide Rotman lens: (**a**) input = F_11_, (**b**) F_22_, and (**c**) F_33_.

Figure 5 shows the simulated electric field of a three-dimensional waveguide Rotman lens. Figure 5a,b and c are for diagonal ports F_11_, F_22_ and F_33_, respectively. The progressive field distribution can be found. 

**Figure 6:**
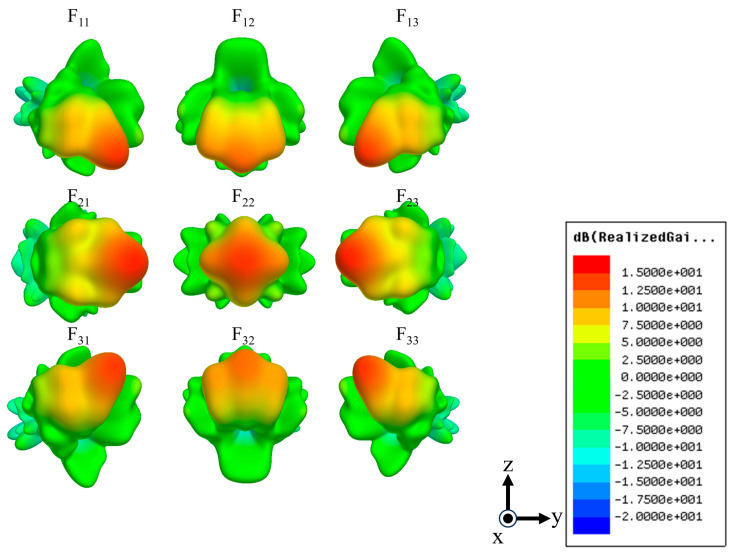
Simulation results for the radiation pattern of the three-dimensional waveguide Rotman lens antenna.

Figure 6 shows the results of simulations of the radiation patterns for all nine input ports. The gains and beam tilt angles are summarized in Table 2. The radiation pattern for each input exhibits symmetrical beamforming based on F_22_ of the central input. The absolute values of the beam tilt angles in the longitudinal, transverse, and diagonal directions are 20°, 25°, and 17°, respectively. F_22_ of the central input port exhibits the highest gain of 9 dBi. F_11_, F_13_, F_31_, and F_33_ of the diagonal Rotman lens have the lowest gains of 1.6 dBi. The beam tilt angle is 5°, i.e., not as designed. There are two reasons for this error. First, the incident wave was delivered from the input waveguide to the free space, not to the output waveguides, during simulation; the radiation pattern combines the radiation power in the free space. The tuning of the waveguide positions may also have been associated with some error. The proposed Rotman lens engages in three-dimensional beamforming at each input port, although the output phase and beam tilt angle contain errors.

The frequency was set to 28 GHz, which is within the commercialized frequency band. For easy fabrication, the number of tilted beams was set to 3 × 3, and the radiating elements were also set to 3 × 3. Considering the number of elements, the tilted beam was aimed at −20°, 0°, and +20°. The feeding port and antenna ports are composed of a metallic open-ended waveguide. The initial spacing between the lateral radiating elements was set to 0.8 of wavelength λ. The initial focal length F was set to 3 λ, and the center of the focal arc G was 1.113 of F. The initial scan angle α was set to 28.4°. After setting the initial parameters, the antenna was optimized by adjusting each parameter. The simulation was performed using Ansys HFSS.

**Figure 7:**
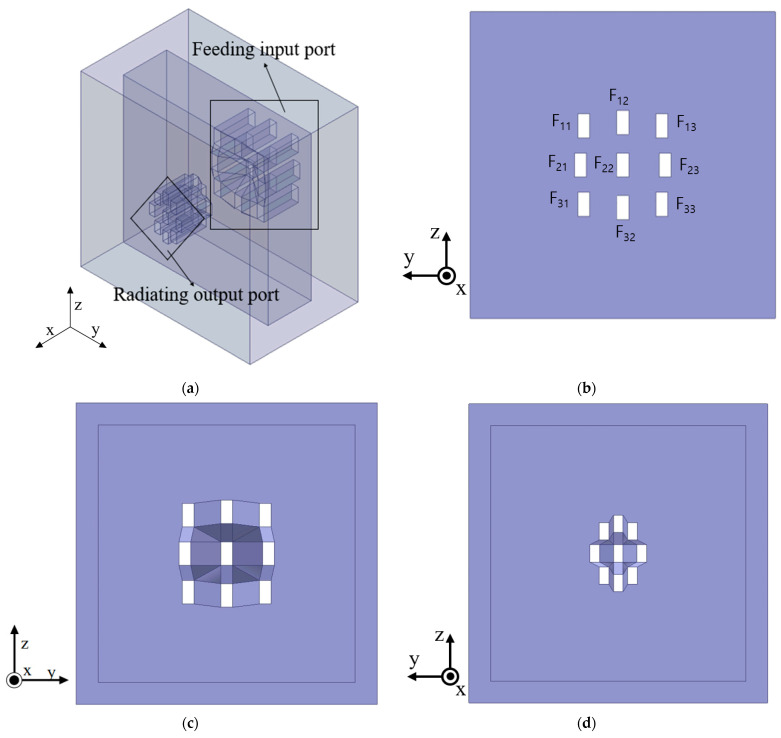
Proposed volumetric Rotman lens antenna: (**a**) perspective view and (**b**) side view; (**c**) inside input and (**d**) inside output.

Figure 7 shows a transparent view of the proposed volumetric Rotman lens antenna. The feeding waveguides are shown on the left, and the radiating apertures (open-ended waveguide antenna) are shown on the right. The aperture of the waveguide follows the general standard of WR-34 of 8.64 × 4.32 mm. The inner spacing of the proposed antenna is filled with air, and the absorbing material is attached to the inner wall to remove the reflected wave from the conducting plate. The input port is indexed, as shown in Figure 7b similar to the Figure 2.

As shown in Figure 7, the widths of the radiating waveguides are slightly different in order to agree with the transmission phase by adjusting the guided wavelength [21]. The width of central waveguide is 6.5 mm, and others are 6.4 mm.

**Figure 8:**
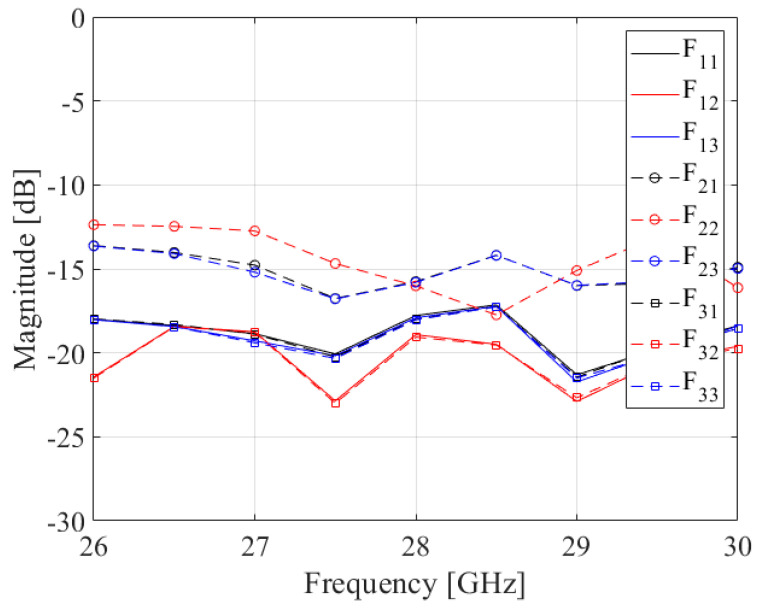
Simulated results of reflection coefficient for each input port.

Figure 8 shows the simulated results. The x-axes are the frequency in GHz and the y-axis is the magnitude of the reflection coefficient. Five curves are drawn for each feeding input port. As shown in the figure, the reflection coefficient is below −15 dB at 28 GHz.

**Figure 9:**
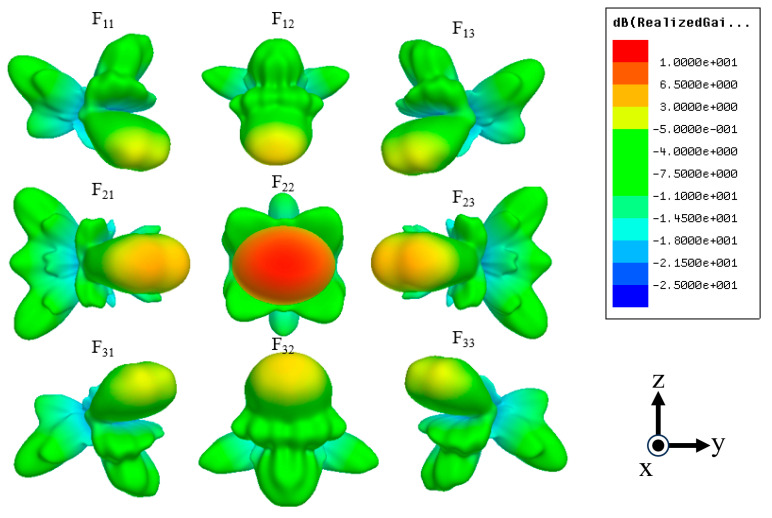
Simulated results of radiation pattern for each input port.

The radiation pattern for each input port was simulated, as shown in Figure 9. As indicated by Figure 9, the beam pattern for the central axis shows the on-axis beam. The direction is tilted depending on the feeding point. In summary, the beam direction in the azimuth and elevation angles (φ, θ) are (−16°, −17°), (0°, −25°), (16°, −17°), (−20°, 0°), (0°, 0°), (20°, 0°), (−16°, 17°), (0°, 25°), and (16°, 17°) for beam indices 1–9, respectively. There are some differences compared with the designed goal, but they are considered acceptable considering the broad beamwidth of 20°.

## 4. Fabrication and Measured Results

**Figure 10:**
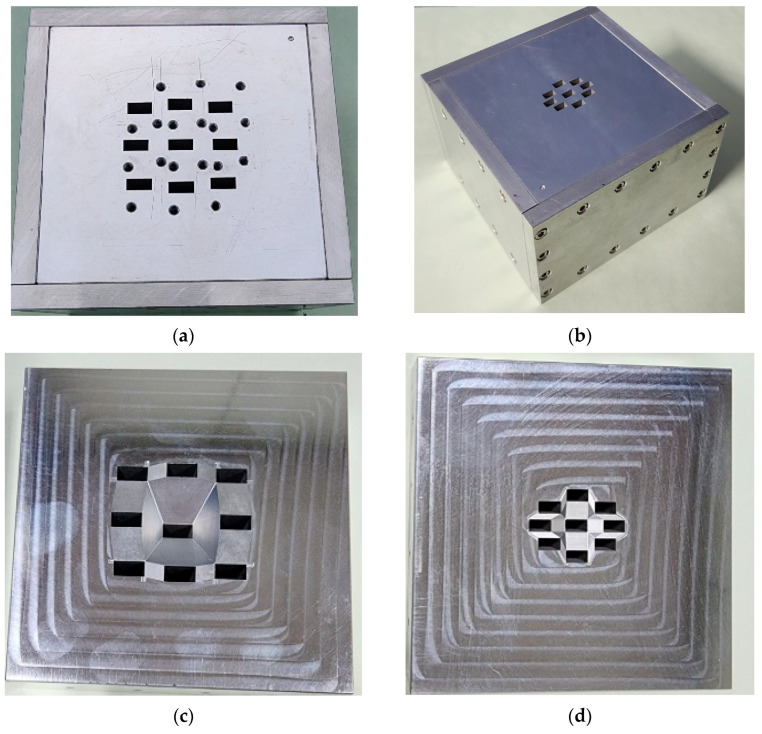
Fabricated volumetric Rotman lens antenna: (**a**) outside view of feeding waveguide, (**b**) outside view of radiation aperture, (**c**) inside view of feeding waveguide, and (**d**) inside view of radiating aperture.

The designed antenna was fabricated as shown in Figure 10. The entire volume was 94 mm × 94 mm × 70 mm, which was just 15.3% of the volume, 155 mm × 72 mm × 362 mm, of the previous two-stage stacked Rotman lens antenna [21]. Six rectangular blocks were made by the milling process and then assembled using a metallic bolt. As shown in Figure 10a, the feeding waveguide was milled into the back plate. The radiating waveguide was drilled into the front plate (opposite side of the back plate). This design can achieve a low fabrication cost. The 3 × 3 feeding waveguide and the 3 × 3 radiating aperture are also shown. The absorber was attached to the wall of the fabricated antenna.

**Figure 11:**
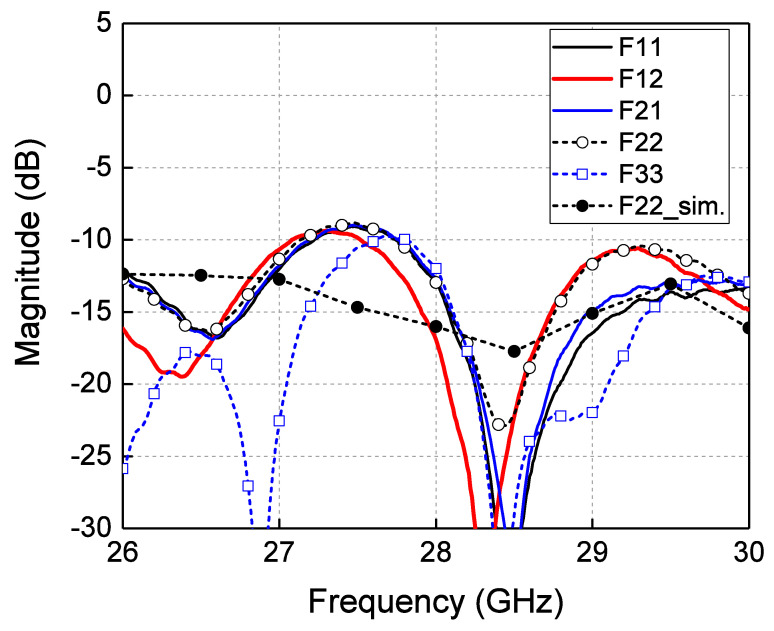
Measured reflection coefficients of the proposed volumetric Rotman lens antenna.

Figure 11 shows the reflection coefficient from the measurements. The measured reflection coefficient was −15 dB for all feeding input ports at 28 GHz. There are differences between the simulated and measured results, which might be from the absorber that is inserted in the waveguide of the Rotman lens.

**Figure 12:**
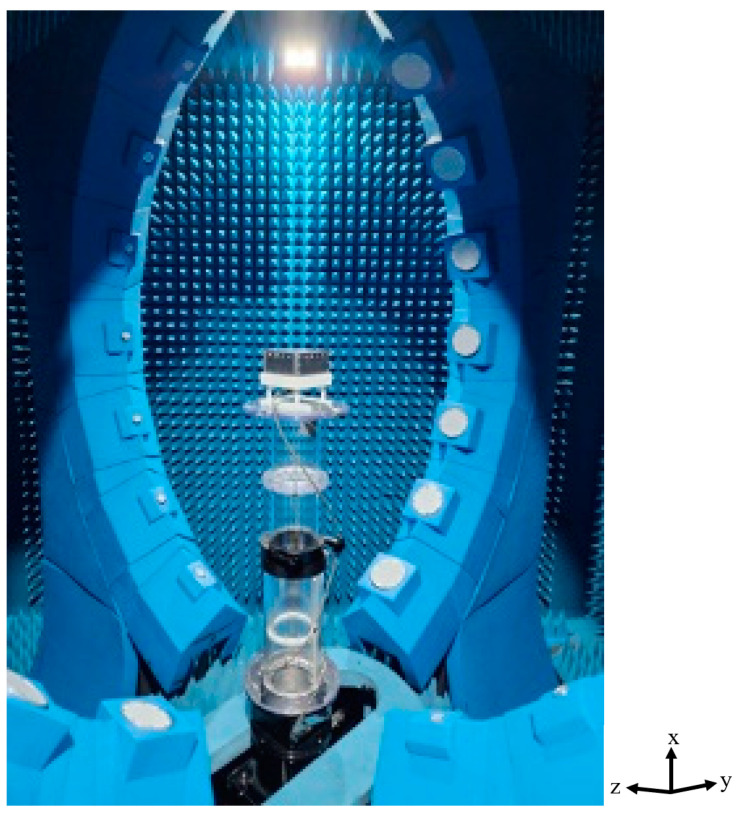
Measurement setup using multiprobe near-field measurement system.

The radiation pattern for each input port was measured using a multiprobe near-field measurement system, as shown in Figure 12 [22].

**Figure 13:**
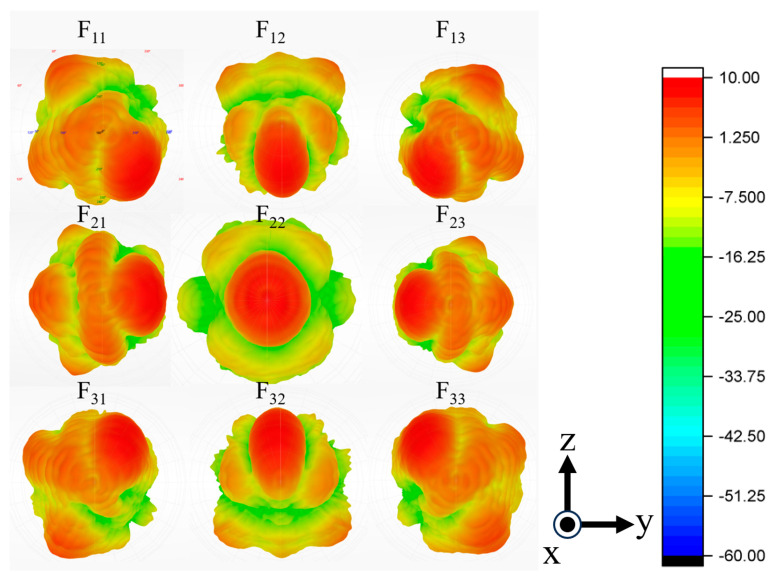
Measurement radiation pattern of fabricated volumetric Rotman lens antenna.

From the experimental results, the beam directions in the azimuth and elevation angles (φ, θ) are as follows: (−17°, −22°), (0°, −22°), (17°, −23°), (−25°, −1°), (−5°, −2°), (26°, 3°), (−15°, 24°), (3°, 26°), and (17°, 24°) for beam indices 1–9, respectively (Figure 13). Similar to the simulated results, the beam pattern for the central axis shows the on-axis beam. The beam-titling angle of the fabricated antenna was almost the same as the simulated results. However, the gain was slightly different. This is because the boundary area between the feeding input ports and radiating output ports was set as a radiation surface with a reflection level of −60 dB. However, in real fabrication, it was implemented as a flat absorber with a −10 dB reflection level. So, the imperfect boundary could interfere with the propagation between the input and output ports. As proof, when a boundary of a −10 dB reflection level is applied in simulation, the simulated gain changes with 4.22, 2.5, 4.27, 6.04, 6.38, 5.99, 4.2, 2.48, and 4.19 dBi for beam id of F_11_, F_12_, F_13_, F_21_, F_22_, F_23_, F_31_, F_32_, and F_33_, respectively. These are different from the simulated gain, as listed in Table 3. This phenomenon could be overcome by the usage of a pyramidal absorber inside the volume of the Rotman lens.

## 5. Conclusions

In this paper, a volumetric Rotman lens antenna that operates at 28 GHz was developed. The design formula and consecutive procedure determining the position of input and output ports of the 3-D Rotman lens antenna were derived, and the closed-form equation was proposed, which to the best of our knowledge might be the first attempt at this. The initial parameter was utilized for the simulation, and then, the optimization process was performed. The ideal case for a 3-D Rotman lens was simulated, and beam-tilting in two directions was confirmed. The real case of fabrication was also simulated, and then, the final design of the antenna was fabricated. The input waveguides were drilled into the back plate, and the radiating aperture was milled into the front plate. The radiation pattern and gain measurements were performed by using the rapid multiprobe near-field system. The gain of the fabricated antenna shows a difference compared with the simulated results, which might be the absorbing material inside the waveguide cavity. In conclusion, it can be said that the proposed 3-D Rotman lens antenna is controlled almost as calculated, and to the best of our knowledge, it might be a first attempt at this. The proposed antenna can reduce the volume by approximately 75%, and the required volume for beamforming is only 50 mm × 50 mm × 35.8 mm. Even though the proposed antenna is heavier compared with the microstrip type, it could be appropriated as an antenna for 5G small-sized communication systems such as a base station or in-door repeater.

## Figures and Tables

**Table 1:** Simulation results of the phase for a three-dimensional waveguide Rotman lens.

Port	*F*_11_	*F*_12_	*F*_13_	*F*_21_	*F*_22_	*F*_23_	*F*_31_	*F*_32_	*F*_33_
*I*_11_	−70.5				−100.6				−56.7
*I*_12_		−19.2			−98.3			26.9	
*I*_13_			−70.5		−99.9		−56.5		
*I*_21_				−42.1	−98.4	50.8			
*I*_22_	135.6	−144.1	135.7	−155.9	−98.3	−156.2	135.6	−143.3	136.3
*I*_23_				51.7	−98	−41.8			
*I*_31_			−56.8		−99.9		−70.5		
*I*_32_		26.7			−98.4			−19	
*I*_33_	−56.7				−100.3				−70.3

**Table 2:** Simulation results for tilt angle and gain.

Beam	Phi (deg)	Theta (deg)	Gain (dBi)
F_11_	20	−17	12.8
F_12_	0	−20	10.9
F_13_	−20	−17	12.8
F_21_	22	0	13.5
F_22_	0	0	12.9
F_23_	−22	0	13.5
F_31_	20	17	12.8
F_32_	0	20	11.0
F_33_	−20	17	12.9

**Table 3:** Beam direction and gain for the simulated and measured results.

Beam	Phi (deg)		Theta (deg)		Gain (dBi)	
	Sim.	Meas.	Sim.	Meas.	Sim.	Meas.
F_11_	−16	−17	−17	−22	1.6	1.8
F_12_	0	0	−25	−22	2.7	3.8
F_13_	16	17	−17	−23	1.5	−0.1
F_21_	−20	−25	0	−1	4.3	2.2
F_22_	0	−5	0	−2	9.0	6.9
F_23_	20	26	0	3	4.2	2.3
F_31_	−16	−15	17	24	1.6	0.3
F_32_	0	3	25	26	2.7	3.8
F_33_	16	17	17	24	1.5	0.2

## Data Availability

The data presented in this study are available on request from the corresponding author due to the successive research.
